# Combined treatment for a rare malignant glomus tumor of the esophagus with pulmonary and liver metastases: a case report and review of literature

**DOI:** 10.3389/fonc.2024.1340859

**Published:** 2024-05-31

**Authors:** Yanan Liu, Jingjing Mao, Dongfeng Shen, Baoli Jin, Xueqin Wu, Congcong Song, Wenjing Du

**Affiliations:** ^1^ Shanxi Province Cancer Hospital, Shanxi Hospital Affiliated to Cancer Hospital, Chinese Academy of Medical Sciences/Cancer Hospital Affiliated to Shanxi Medical University, Taiyuan, Shanxi, China; ^2^ Department of Translational Medicine, Shenzhen Engineering Center for Translational Medicine of Precision Cancer Immunodiagnosis and Therapy, YuceBio Technology Co., Ltd, Shenzhen, China; ^3^ Department of Tumor Minimally Invasive Therapy, Shanxi Traditional Chinese Medical Hospital, Taiyuan, Shanxi, China

**Keywords:** malignant glomus tumor, esophageal, anlotinib, immunotherapy, case report

## Abstract

**Background:**

Glomus tumors are typically benign soft tissue tumors that occur at the extremities; malignant and viscerally occurring cases are extremely rare.

**Case presentation:**

We report a 49-year old male patient with a malignant esophageal glomus tumor that was complicated by lung and liver metastases. Genetic test results guided the patient’s individualized treatment. Consequently, treatment with Anlotinib combined with Tislelizumab achieved significant clinical benefits.

**Conclusion:**

Our case report demonstrates that immunotherapy combined with anti-angiogenic therapy in patients with malignant esophageal glomus tumors can achieve significant efficacy and suggests the potential value of next-generation sequencing (NGS) detection in guiding personalized treatments in patients with malignant esophageal glomus tumors.

## Background

Glomus tumors(GT) are uncommon mesenchymal neoplasms mainly derived from perivascular modified smooth muscle cells, the majority of glomus tumors occurs in the skin and superficial soft tissues of the distal extremities, at a site other than the limbs are extremely uncommon (1–3). Most GTs are benign, malignant glomus tumors are extremely rare accounting for <1% of all glomus tumors. Only a few cases of malignant GTs have been reported, and most of them were locally aggressive and distally metastatic (3, 4). The diagnosis of malignancy should take into account tumor size, infiltration, mitotic activity, nuclear atypia, and vascular involvement (5). By summarizing the pathological features of 52 cases, Folpe and colleagues proposed a subclassification of atypical and malignant GTs (6). The criteria for the diagnosis of malignancy are as follows: i) deep location of the tumor, ii) size of the tumor>2cm, iii) atypical mitosis or obvious nuclear heterogeneity, iv) mitotic cells accounting for five or more of 50 under high power field (HPF). If a GT is larger than 2cm and deep in location, but without nuclear heterogeneity, it will be classified as an undetermined malignant potential GT (7).

They usually present clinically as a triad of severe subungual pain, tenderness localized over a point, and cold hypersensitivity (8, 9). The optimal treatment of malignant glomus tumors remains unknown, and the current published literature consists of individual case reports or series, local wide excision remains the most viable treatment option.

In this report, our objective was to present a unique case of a malignant esophageal glomus tumor with pulmonary and liver metastases. After treatment, the patient’s condition has improved, his quality of life has significantly improved, and their life has also been extended.

## Case presentation

A 49-year-old Chinese male initially presented with dysphagia and foreign body sensation without obvious inducement. Later, the patient presented with aggravated dysphagia accompanied by chest and back pain; therefore, he was treated in our hospital. A non-enhanced chest computed tomography (CT) scan showed a large esophageal tumor, and the mass size measurement was around 4.6 × 3.9 × 8.3 cm and located in the middle part of the esophagus. One month later, the patient was admitted to Tianjin Cancer Hospital. CT and endoscopic ultrasound confirmed that the patient had Stage T4 esophageal cancer with multiple mediastinal lymph node enlargements. The patient underwent surgery under general anesthesia with three incisions: radical resection of the lower thoracic segment of the esophagus, partial gastrectomy, tube and gastroplasty, left cervical esophagogastrostomy, thoracic and abdominal lymph node dissection, and jejunostomy. Postoperative pathological and immunohistochemical detection results indicated that the patient had a malignant glomus tumor in the middle section of the esophagus, and no tumor metastasis was observed in the regional lymph nodes. The final diagnosis was confirmed by immunohistochemistry of positive smooth muscle actin (SMA), vimentin, B-cell lymphoma-2 (Bcl-2), and a Ki67 index of 40%. The specimen showed negative staining for CK5/6, CK8/18, P40, S-100, CD34, ERG, EMA, CD31, Desmin, CD117, and LCA. The patient was not treated postoperatively.

The disease progressed by four months after surgery (December 17, 2021). CT examination revealed submediastinoid nodules and multiple nodules in both lungs, and metastasis was considered. January 1, 2022, the patient then underwent chemotherapy with docetaxel and cisplatin for one cycle. Docetaxel 100mg, ivgtt, d1, cisplatin 30mg, ivgtt, d1-3, repeated every 21-28 days. However, cough, hemoptysis, and obvious chest and back pain were observed after one cycle chemotherapy, and symptomatic treatment for medical hemostasis was ineffective.

**Figure 1:**
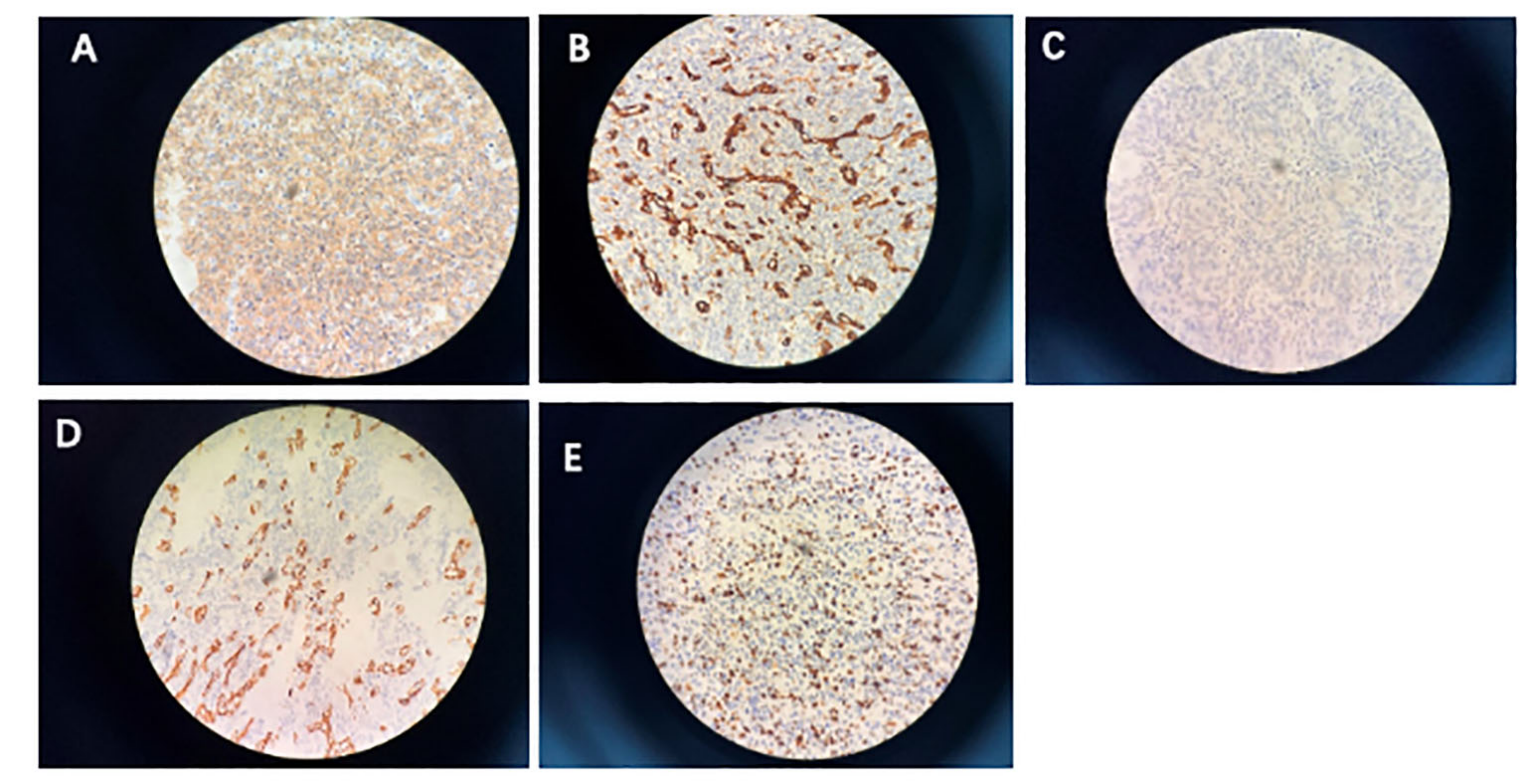
Immunohistochemistry. **(A)** Smooth muscle actin; **(B)** CD31; **(C)** caldesmon; **(D)** CD34; **(E)** proliferative index (Ki-67) is approximately 60%.

On February 10, a follow-up examination showed the progression of bilateral lung nodules and liver metastases. At this time, there were nearly 20 bilateral lung metastases, with the largest one having a diameter of 1.2cm. There were multiple liver metastases, with the larger one located in the left lobe of the liver, approximately 3.1 * 2.0cm. Percutaneous selective arteriography was performed under local anesthesia on February 15, 2022. After percutaneous arterial embolization, the hemoptysis was slightly relieved. CT results on March 1 again showed multiple pulmonary nodules that were larger than before, with the diameter of the largest nodule increasing to 1.2 cm. An irregular mass was found near the left side of the chest and stomach, with a size of 4.3 × 2.7 cm, which compressed the left main bronchus, a large amount of fluid in the left thoracic cavity, and abdominal lymph node metastasis, considering the progression of the disease. The patient coughed violently, expelling soft tissue-like masses. These masses were examined pathologically and combined with immunohistochemical results, showed positivity for CD31, SMA, Caldesmon, CD34, and the Ki67 index was 60% (**Figure 1**). Combined with the history, the presentation was consistent with a malignant glomus tumor.

We combined the results of genetic testing to provide the patient with systemic therapy. NGS test results revealed a mutated gene, KEAP1, associated with immunosuppressive resistance at a high frequency, and the mutation abundance was 29.16%. Therefore, the patient was treated with simultaneous radiochemotherapy: radiotherapy for an irregular mass on the left side of the thoracic stomach, DT: 58 Gy/29 times/43 days, during synchronous paclitaxel (albumin type) (100 mg) weekly treatment four times for a total of 400 mg. CT review 20 days after the final treatment showed that the left lung was re-expanded, and the left pleural effusion was significantly reduced. However, multiple solid nodules in both lung fields were larger than before, and multiple liver metastases were observed on May 10, 2022. The disease progressed rapidly, and Anlotinib targeted therapy was intended, but the patient was unable to tolerate intermittent hemoptysis.

To further control hemoptysis, the patient underwent implantation of 60 I125 radioactive particles into the subcarina mass on June 6, 2022. CT reexamination 20 days after surgery showed that the irregular mass in the left side of the chest and stomach was further reduced. The lesions in both lungs and liver have further progressed, with nearly 30 metastases in both lungs and significantly larger than before, with the largest being approximately 2.1 * 3.6cm. Liver metastasis has also increased compared to before, with the larger one being about 5.9 * 4.3cm. At this time, the patient’s general condition is good, and there is no obvious cough, sputum, or hemoptysis. Starting from June 30, 2022, oral administration of anlotinib hydrochloride capsules 12mg, Qd, d1-14, repeated every three weeks. But after 10 days of oral administration, the patient intermittently had blood in the sputum with bright red color, so oral administration of anlotinib hydrochloride capsules was suspended and anti infection and hemostatic treatment was given. On July 15, 2022, a follow-up examination showed that the lung lesion had partially shrunk, with the maximum metastatic lesion shrinking from 2.1 * 3.6cm to 1.8 * 2.8cm, and the maximum liver metastatic lesion shrinking from 5.9 * 4.3cm to 5.3 * 3.5cm. The symptom of blood in sputum disappeared, and the initial treatment was shown to be effective.

**Figure 2:**
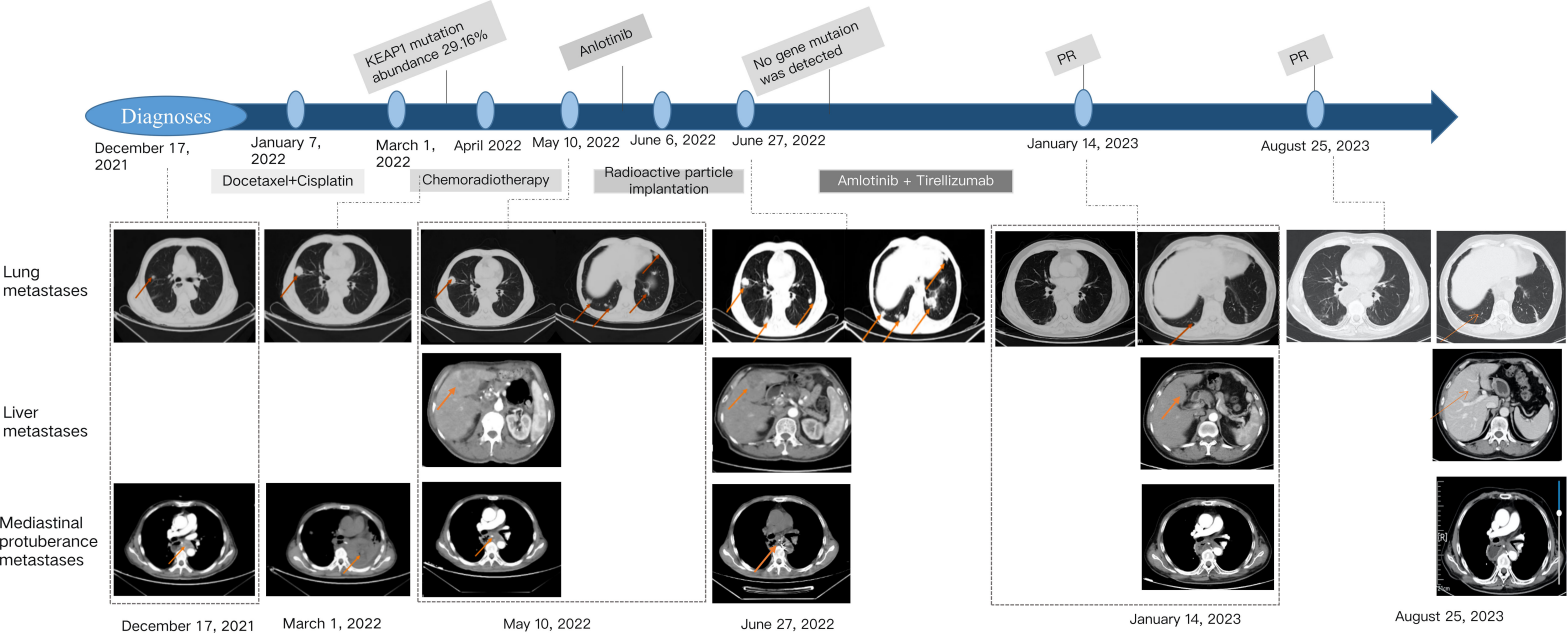
Course of the disease with treatment history and images.

Therefore, we once again considered precision treatment for the patient based on the results of genetic testing, which showed that the KEAP1 gene was no longer detected. At this time, the patient’s hemoptysis symptoms disappeared. After the patient’s cough and hemoptysis symptoms improved, the treatment was restarted on October 17, 2022, with the combination of anlotinib hydrochloride capsules and tislelizumab. The specific dosage was anlotinib 10mg, po, d1-14, tislelizumab injection 200mg, ivgtt, d1, repeated every three weeks. On January 14, 2023, a follow-up examination showed significant reduction in both lung nodules and multiple liver nodules compared to before, once again confirming the good therapeutic effect. According to RECIST 1.1, the efficacy was evaluated as a partial response (PR). After that, the patient continued the intermittent treatment of the scheme for 4 cycles (the treatment was interrupted due to repeated infection of COVID-19 and intermittent hemoptysis), and the last treatment was on June 12, 2023. The latest follow-up and reexamination date is August 25, 2023, indicating that the intrahepatic metastasis has further shrunk, with the largest being about 1.1 * 1.5cm in size, the efficacy was evaluated as PR (**Figure 2**). No significant changes were observed in the remaining lesions, and no treatment was taken before leaving the hospital. The patient maintained a PR for more than 7 months. Unfortunately, on September 19, 2023, the patient experienced vomiting blood and gastroscopy revealed perforation of the residual stomach. Despite receiving active supportive treatment, the patient passed away on October 15, 2023 due to gastrointestinal bleeding.

## Discussion

The optimal treatment of malignant glomus tumors remains unknown, especially for patients with metastasis. To date, 16 cases of malignant glomus tumors of esophageal origin have been reported, 7 of these cases exhibited regional or distant metastasis (**Table 1**) (2, 10–22). Due to the rarity of this disease and scarcity of case reports, we have summarized the three cases of malignant hematoma of the esophagus. Zhang et al. (15) reported the first case of malignant hematoma of the esophagus in a 47-year-old man with local lymph node involvement who underwent surgical resection. At the time of publication, there was no evidence of tumor recurrence 11 months after surgery. Seban et al. (20) reported a second case in a 45-year-old man with fluorodeoxyglucose-avid malignant esophageal hemangioma with multiorgan invasion that spread to the mediastinum, liver, scalp, and pelvis. The patient underwent subcutaneous scalp nodule excision, followed by doxorubicin and pelvic external-beam radiation therapy (EBRT). FDG-positron emission tomography results showed a partial metabolic response in the lung. Pazopanib was later introduced, and a complete metabolic response in the liver and a partial response in the bone and mediastinum were achieved. It was discontinued after three months owing to side effects. Despite stopping treatment, continuous imaging showed progress. Subsequent treatment with Regrafenib, cyclophosphamide, and pelvic EBRT exhibited no response and was poorly tolerated. Xiao et al. (1). reported a third case involving a 57-year-old Hispanic woman with malignant esophageal hematoma and lung metastasis. The patient was treated with palliative radiotherapy while waiting for the patient’s NGS to reveal fusion of NOTCH2 (MIR143-NOTCH2). Therefore, a trial of gemcitabine and docetaxel was planned for the patient, but the patient experienced rapid progression and pain after receiving a single dose of gemcitabine, and eventually chose hospice care. Papke et al. (21) reported the fourth case involving a 61-year-old man with malignant esophageal malignant glomus tumor and brain, lung, bones metastasis, but the treatment and prognosis information was not reported. Lastly, Birkness-Gartman et al. (2) reported 3 metastatic esophageal glomus tumor. Patient 1 was a 65-year-old male, he developed metastases to the lung and pericardium 6 years after diagnosis, and was alive at last clinical follow-up (9 years). Patient 2 was a 19-year-old femal developed metastatic disease to the scalp 11 months after resection of her primary tumor, she late developed paraesophageal soft tissue recurrence and metastases to the pleura, pericardium, and diaphragm. She received adjuvant treatment with several different regimens (doxorubicin, ifosfamide,pazopanib, and radiation; nirogacestat; axitinib and pembrolizumab) and was alive at last clinical follow-up (5 years). Patient 3 was a 62-year-old femal, she had a large tumor abutting the aorta on imaging. Intraoperatively, the tumor was adherent to the aorta, resulting in a difficult operation. This patient developed an aorto-esophageal fistula 20 days after surgery, resulting in a fatal hemorrhage, total survival time following diagnosis was 4 months. Our case report can enrich the clinical data of malignant esophageal glomus tumors, and this case is the first malignant esophageal glomus tumors case to receive immunotherapy, providing a clinical basis for the treatment of rare malignant glomus tumors.

**Table 1:** Review of available case reports of malignant esophageal glomus tumors.

Case No.	Reference (author, year)	Sex	Age	Metastatic site(s)	Intervention	Status at time of publication
1	Rueff, 1967 (10)	Unknown	Unknown	Unknown	Unknown	Unknown
2	Utkin, 1972 (11)	Unknown	Unknown	Unknown	Unknown	Unknown
3	Papla, 2001 (12)	Female	79	Unknown	Unknown	Unknown
4	Altorjay, 2003 (13)	Unknown	Unknown	Unknown	Unknown	Unknown
5	Tomas, 2006 (14)	Female	28	None	Surgery	Alive; NER at 6 months
6	Zhang, 2013 (15)	Male	47	Lymph node	Surgery	Alive; NER at 11 months
7	Bali, 2013 (16)	Female	49	None	Surgery	Alive; NER at time of publication
8	Segura, 2015 (17)	Female	66	None	Surgery	Alive; NER at time of publication
9	Ugras, 2015 (18)	Female	47	None	Surgery	Alive; NER at 10 months
10	Marcella, 2019 (19)	Male	30	None	Surgery	Alive; NER at 1 year
11	Seban, 2020 (20)	Male	45	Liver, lung, mediastinum, bone, skin	Surgery, chemotherapy, EBRT	Unknown; progressive disease
12	Xiao, 2022 (21)	Female	57	Lung, lymph node	Palliative IMRT	Alive; progressive disease on hospice
13	Papke, 2022 (22)	Male	61	Brain, lung, bones	Unknown	Unknown
14	Birkness-Gartman, 2023 (2)	Male	65	Lung, pericardium	Unknown	Alive with disease (9 years)
15		Female	19	Scalp, pleura, pericardium, diaphragm	doxorubicin, ifosfamide, pazopanib, and radiation	Alive with disease (5 years)
16		Female	62	Lymph node	Surgery	Died of disease (4 months)

Our case was diagnosed as malignant hematoma of the esophagus complicated with lung and liver metastases. No significant curative effect was achieved with radiotherapy or chemotherapy, and the disease progressed rapidly. Therefore, we attempted to implement a more precise treatment strategy. The patient underwent NGS using the Yuce biological solid tumor clinical drug 1012+PD-L1 | YuceOne^®^ Pro+. This technology has already been used clinically as an effective tool to create personalized treatment plans. Glomus tumors have been reported to have the highest tumor mutation burden among soft tissue sarcomas, with 16% of participants detecting mutations associated with FDA-approved treatments or drugs in development (22). However, the tumor molecular signatures of our patient did not indicate relevant targets that could be directly affected, but there was a gene that was negatively correlated with immunotherapeutic efficacy – KEAP1 (23). KEAP1 mutations confer worse outcomes to immunotherapy among lung cancer patients with KRAS mutation, and KEAP1 mutations results in distinct immunophenotypes in KRAS mution of lung cancer (24). Among atezolizumab and/or bevacizumab with carboplatin/paclitaxel (CP) chemotherapy clinical trial, KEAP1 mutations were associated with inferior OS and PFS across treatments compared with KEAP1-WT (25). KEAP1 mutation can affect the tumor immune microenvironment and cause immune escape. One lung squamous cell carcinoma study showed that KEAP1 mutation was associated with dramatically lower CD8+ TIL density (P = 0.005) (26). KEAP1 mutation in lung adenocarcinoma reduces the dendritic cell and T cell responses that drive immune therapy resistance, leading to immune resistance in lung adenocarcinoma patients (27). This finding suggested against using immunotherapy in this patient. Anlotinib belongs to a class of small-molecule, multi-target anti-angiogenic drugs that have been approved for vesellar soft tissue sarcoma, clear cell sarcoma, and other advanced soft tissue sarcomas that have progressed or recurred (28–30). This drug is contraindicated in patients with a high risk of hemoptysis. Therefore, local radioactive particle implantation was first performed for the patient to relieve the symptoms of hemoptysis, and the subsequent improvement of the patient demonstrated that this step played a key role in the overall treatment of the patient, which also reflected the obvious benefits of multidisciplinary treatment. It has been reported that radiotherapy and chemotherapy can change the immune microenvironment of patients (30–33). Subsequent use of the Yuce biological solid tumor clinical drug 1012+PD-L1 | YuceOne^®^ Pro+, in order to find the targets of effective drugs, showed that no genes negatively related to immune efficacy were detected. Therefore, it can be speculated that the radioactive particle implantation changed the immune microenvironment of the patient. It has been suggested that anti-drugs and PD-1/PD-L1 inhibitors can jointly act on the tumor microenvironment, reshaping the tumor vascular and immune microenvironments, transforming the immunosuppressive state into the immune-promoting state, increasing the invasion of T cells to the tumor, and playing a synergistic anti-tumor effect (1 + 1>2) (34, 35). Anlotinib in combination with immunotherapy has also been reported to significantly improve the prognosis of patients with liver cancer (36). Therefore, we treated the patient with anlotinib combined with Tislelizumab, which showed significant efficacy, significant reduction of intrahepatic lesions, physical improvement, and weight gain of the patient upon follow-up.

The optimal treatment for malignant hematocrystomas of the esophagus remains unclear. A review of published literature shows that extensive local resection remains the most feasible treatment. Mixed success with chemotherapy has also been reported in a small number of cases (15, 37). Our case demonstrates the malignant potential and high aggressiveness of malignant hematoma of the esophagus. Changes in the patient’s immune microenvironment were detected by NGS, guiding the targeted therapy of the patient. This suggests that NGS detection can develop an effective personalized treatment plan for this patient group and has great potential for successful treatment of malignant esophageal glomus tumors in the future.

## Data availability statement

The original contributions presented in the study are included in the article/supplementary material. Further inquiries can be directed to the corresponding author.

## Ethics statement

The studies involving humans were approved by Shanxi Province Cancer Hospital. The studies were conducted in accordance with the local legislation and institutional requirements. The participants provided their written informed consent to participate in this study. Written informed consent was obtained from the individual(s) for the publication of any potentially identifiable images or data included in this article.

## Author contributions

WD: Conceptualization, Data curation, Funding acquisition, Writing – review & editing. YL: Conceptualization, Writing – original draft. JM: Writing – original draft. DS: Data curation, Investigation, Writing – review & editing. BJ: Data curation, Formal analysis, Writing – review & editing. CS: Writing – review & editing, Validation. XW: Data curation, Writing – review & editing.
